# Microwave-assisted rapid conjugation of horseradish peroxidase-dextran aldehyde with Schiff base reaction and decolorization of Reactive Blue 19

**DOI:** 10.55730/1300-0527.3378

**Published:** 2022-02-25

**Authors:** Mithat ÇELEBİ, Zafer Ömer ÖZDEMİR, Murat TOPUZOĞULLARI

**Affiliations:** 1 Department of Polymer Materials Engineering, Yalova University, Yalova, Turkey; 2 Department of Analytical Chemistry, University of Health Sciences Turkey, İstanbul, Turkey; 3 Department of Bioengineering, Yıldız Technical University, İstanbul, Turkey

**Keywords:** Conjugation, decolorization, gel permeation chromatography (GPC), microwave irradiation, peroxidase, reactive dye

## Abstract

Microwave irradiation has become a routine technique in homogeneous and effective heating in organic synthesis. However, its application in enzyme-containing reactions is limited since it can cause denaturation of the enzyme. In this study, we have briefly investigated the effect of microwave heating on the conjugation reaction of horseradish peroxidase (HRP) with aldehyde derivative of dextran (D-CHO). The reaction was irradiated by microwave at 50 °C for 5 min. The conjugate was confirmed via GPC, in which the conjugates of HRP and D-CHO coexist with free unbound HRP molecules. Activity studies of HRP revealed that there is a small decrease in conjugate activity relative to the free enzyme after a short bioconjugation reaction with microwave irradiation. In decolorization studies of the textile dye Reactive Blue 19 (RB19), 99% of RB19 was decolorized through the free enzyme at 35 °C while the decolorization of the dye was 96% at 25–35 °C by the conjugate, which is a critical result showing clearly that the HRP conjugated via D-CHO is not denatured and still active after microwave-assisted reaction. This phenomenon is due to the multiple point conjugation of D-CHO on the surface of HRP and locking the 3D structure which may prevent changes in the secondary or tertiary structure of the enzyme. The results reveal that microwave irradiation can be used in production of covalently modified enzymes.

## 1. Introduction

Microwave-assisted synthesis is presently a big concern in organic synthesis to promote bioconjugation reactions, although microwave irradiation has gained importance in the past decades as a powerful tool for the rapid and efficient chemical synthesis of a variety of compounds [1–6]. Microwave-assisted irradiation employs the power of active electric charges exhibiting in solution or conducting ions in solid to convert electromagnetic energy into heat. Direct heating of molecules by microwave energy provides a homogeneous product within a very short time, less by-products and higher efficiency in the reactions. Microwave-assisted reactions occur against dielectric heating; that is, particles demonstrating a constant dipole moment attempt to adjust to the used electromagnetic field, which causes the molecules to rotate, increase friction, collide, and hence heat production [7,8]. The fast dielectric orientation of the dissolving agent and the reacting substance under microwave irradiation causes shortened chemical reaction periods, enhanced performance, and refinement of the products [9]. Also, microwave irradiation can be used for enzymatic reactions and enzymatic hydrolysis in water for biological sciences [10,11]. However, the use of microwave radiation in the reactions for modification of enzymes has been limited due to microwave denaturation of the enzymes [12].

Horseradish peroxidase (HRP, EC 1.11.1.7) is an industrially important enzyme used in a variety of applications for environmental protection such as wastewater treatment and bioremediation [13]. Peroxidases (EC 1.11.1.x) can be used to oxidize and degrade toxic dye molecules for the treatment of industrial wastewater [14]. Enzymatic decolorization is one of the methods used to remove dyes, especially from the wastewater of the textile industry. HRP can also be used to degrade expeditiously aromatic azo dyestuffs by the addition of hydrogen peroxide [15]. However, the application of HRP in industrial wastewater is limited, since its activity is highly sensitive to environmental conditions such as pH, temperature, and ions due to the proteinaceous structure of HRP. To overcome this obstacle, the structure of the enzyme can be modified by a various group of molecules such as polymers [16] or small molecules [17].

Enzymes can be modified with polymers to gain desired properties for the development of enzyme-based industrial applications. The use of polymers in the modification of enzymes is very effective since various methods such as encapsulation, complexation or covalent conjugation can be used [18–20]. HRP has also been modified via polymers using the techniques of covalent conjugation with polymers [21] or encapsulation in hydrogels [22]. In the study of Bilal et al., HRP was immobilized in alginate-chitosan hydrogel to degrade the textile dye of RB19 [22]. It was shown that encapsulation of HRP in the hydrogel extended the working pH and temperature range of the enzyme in the decolorization of RB19. HRP was also immobilized on several matrices to decolorize synthetic dye solutions and 4-chlorophenol for wastewater treatment [23]. Efficient and environmentally friendly methods are required to remove toxic dye molecules such as RB19 from the environment and wastewater [24,25].

In the study, the effect of microwave irradiation on the conjugation reaction and the enzymatic activity of HRP was investigated. For this purpose, dextran was oxidized to its aldehyde form and conjugated by HRP under microwave irradiation by forming Schiff base linkages. Then, the conjugate was characterized and its decolorization activity against RB19 was examined at different temperatures. We consider that this study gives briefly critical information about the application of microwave-assisted synthesis to the conjugation of an enzyme and a polymer conjugate.

## 2. Materials and methods

RB19 (M_w_: 624.54 g/mol^–1^), sodium borohydride, sodium meta-periodate, and dextran (M_w_: 60–90 kDa) were purchased from Sigma Aldrich. HRP (M_w_: 40 kDa, catalogue no: 77332) and o-dianisidine were obtained from Fluka. All compounds were used without any further purification. Milestone MicroSynth Microwave Labstation for Synthesis was used for microwave irradiation of the samples. UV-VIS spectroscopy experiments were carried by using a Shimadzu UV-1800 spectrophotometer. Gel permeation chromatography (GPC) studies of HRP and the conjugate were obtained using a Viscotek TDA 302 GPC system with triple detectors. Shim-Pack Diol 300 column was used as the GPC column. Mobile phase was PBS (pH: 7.4) and flow rate was 1.0 mL/min. Telstar Cryodos freeze-dryer system was used to lyophilize the samples.

### 2.1. Oxidation of dextran

Firstly, dextran was reacted with sodium meta-periodate at 25 °C for 24 h to obtain dextran aldehyde (D-CHO). Then, D-CHO was dialyzed (MWCO: 10 kDa) against water to eliminate by-products such as formaldehyde [26]. Finally, the dialyzed D-CHO was lyophilized for characterization and conjugation reaction.

### 2.2. Synthesis of conjugate

In this study, HRP was used without any purification. The molar ratio of HRP and D-CHO in the conjugation reaction was used as n_HRP_/n_D-CHO_: 1/10, in which n_HRP_ is the number of moles of HRP and n_D-CHO_ is the number of moles of D-CHO, respectively. D-CHO was dissolved in 40 mL of distilled water at pH 7.0 (100 mM PBS) and the enzyme was added to this solution with a final enzyme concentration of 1.0 mg/mL. The solution was irradiated in two periods of 5 min at 50 °C with 500 W of microwave energy.

After synthesis, double bonds in the Schiff base groups of the conjugate were reduced using the method in our previous study [26]. Briefly, the pH of the reaction solution was increased to 8.5 by adding sodium bicarbonate, and the solution was stirred for 15 min. Then, sodium borohydride was added at 4 °C for reduction of Schiff base double bonds and incubated for 15 min. Finally, pH of the solution was adjusted to 7.0.

### 2.3. Activity

A stock HRP solution was prepared in 1.0 mg/mL concentration. The final concentration of HRP in HRP/D-CHO conjugate was 0.753 mg/mL. Activities of the HRP and HRP-Dextran Aldehyde Conjugate (HRP/D-CHO) were determined by using the following steps at pH 5.0 and 30 °C. Buffer solution of 960 μL (0.05 M, pH: 5.0), 20 μL o-dianisidine, and 10 μL HRP or HRP/D-CHO solution with equal enzyme concentrations was added to a quartz cuvette and the cuvette was stirred with an orbital shaker. Finally, 10 μL H_2_O_2_ was added to initiate the reaction and then, OD_460_ of this solution was acquired at each 10^th^ min. Activities of the enzyme and the conjugate were calculated by using Equation 1 [27]. In Equation 1, U is the unit of enzyme activity as 1 μmol of o-dianisidine produced in 1 min, A_460_ is the absorbance of the solution at 460 nm, M_e_ is the molar absorption coefficient of o-dianisidine (11.300).


(1) $$\frac{U}{mg}=(\frac{A_{460}\times \;10^{6}}{M_{ɛ}\times C_{HRP}})$$

### 2.4. Decolorization

Synthetic dyestuff wastewater of Reactive Blue 19 (RB19, 40 mg/L) was prepared with a concentration of 40 mg/L at pH 5.0. Decolorization of RB19 was measured by acquiring the absorbance of dye solution at the wavelength of 594 nm at 25 °C for 1 h. Dye solution and enzyme or conjugate were added to the 3.5 mL spectrometry cuvette, respectively. Then, the decolorization reaction was started by the addition of H_2_O_2_ (3%).

Percentages of dye decolorization values were calculated by using Equation 2, in which A_b_ is the absorbance of dye solution before initiation of the enzyme reaction and A_a_ is the absorbance of dye solution at a predetermined time after initiation of enzyme reaction.


(2) $$(\%)Decolorization\;of\;Dyestuff=\frac{A_{b}-A_{a}}{A_{a}}\times 100$$

## 3. Results

The study aims to propose a microwave assisted synthesis method for bioconjugation of HRP and aldehyde derivative of dextran. For conjugation of the dextran and HRP, Schiff base formation was chosen as the conjugation reaction, in which aldehyde groups of the modified dextran (D-CHO) react with amino groups of the enzyme. The GPC with triple detection system was used to characterize chemical and physicochemical structure of the conjugates. Then, enzymatic activity of free and conjugated enzyme was examined to evaluate the efficiency of the method. For this purpose, a toxic textile dye of RB19 was chosen as the substrate.

### 3.1. Characterization of the conjugate

**Figure 1:**
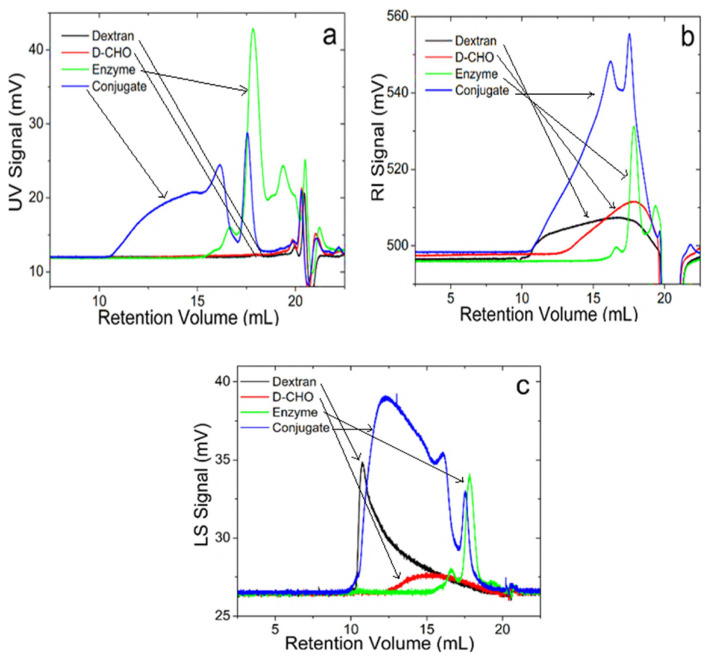
GPC chromatograms of dextran, aldehyde derivative of dextran (Dextran-CHO), HRP, and the conjugate of HRP/D-CHO. **a:** acquired from UV-280 nm, **b**: acquired from RI, **c**: acquired from LS detectors.

The prepared D-CHO was conjugated with HRP by irradiating to 50 °C for 5 min using 500 W of microwave energy. The conjugate was characterized by a GPC system with triple detectors of UV, refractive index (RI), and right-angle light scattering (LS). This equipment allows us to visualize the composition of the macromolecular mixtures and to characterize each component separately. Figures 1a, 1b, and 1c show the GPC chromatograms of the samples acquired from different detectors.

Figure 1a. displays the GPC chromatograms acquired from UV detector which is a distinguishing detector for proteins and polysaccharides since polysaccharides do not absorb the UV wavelengths that the proteins absorb. As seen in Figure 1a, dextran and D-CHO do not have elution peaks in the UV detector except the injection peaks eluted after 19 mL. This is directly related to the very low absorptivity of dextran and D-CHO at 280 nm. On the other hand, HRP has two elution peaks at 16.6 and 17.8 mL. When HRP is conjugated with D-CHO, a new broad peak appears in the chromatogram between 10.5–15 mL, and the peak belonging to free HRP also elutes after this broad conjugate peak. The UV detector signal is directly related to an enzyme’s absorptivity. Therefore, peak areas in the UV chromatogram of the conjugate allow calculating the yield of the reaction in which 75% of HRP is conjugated to the D-CHO.

RI detection is sensitive to the refractive index of the solute and the concentration and therefore can detect almost any molecule. Signals obtained from the LS detector are related to concentration and the molecular weight of the macromolecule. Therefore, these two detection systems can detect any molecule in GPC and are used together to calculate the molecular weight of the macromolecules in the mixture. Figure 1b and 1c show the RI and LS chromatograms of the samples. As seen from Figure 1, dextran and D-CHO have different elution profiles and molecular weights (M_w_) of dextran and D-CHO are 62.5 and 10.1 kDa, respectively. The lower molecular weight of D-CHO compared with dextran can be the result of the degradation of dextran chains after oxidation. HRP gives similar peaks in RI and LS chromatograms to UV chromatograms and their molecular weights were determined as 40.2 and 80 kDa for the peaks eluted at 16.6 and 17.8 mL, which corresponds to monomeric and dimeric HRP molecules due to the clear evidence of molecular weights. Multimer formation of enzymes or proteins can be observed after dissolution in aqueous solutions [28]. The conjugate has two distinct peaks eluted between 10–17.5 mL and at 18 mL in which the peak eluted earlier corresponds to the conjugate due to the formation of larger macromolecular structures after the covalent association of D-CHO and HRP. Moreover, the molecular weight of the conjugate eluted between 10–17.5 mL is 75 kDa and the peak eluted at 17.8 mL is 40 kDa, which directly shows that conjugate and free HRP exist together in the solution. The molecular weight of the conjugate reveals that more than one D-CHO molecules are bound with one HRP molecule. Consequently, GPC chromatograms directly expose the successful conjugation between D-CHO and HRP, but some HRP molecules are unbound by the D-CHO. Compared with the literature, the reaction yield of the conjugation with microwave irradiation is much higher than the conventional reaction between oxidized polysaccharide and enzyme [29].

### 3.2. Enzymatic activity and decolorization

The enzymatic activity is sensitive to environmental changes and to the chemical modifications in the structure of the enzyme because all these changes can directly affect the 3D shape and structure of the enzyme. Therefore, after conjugation, the activity of the conjugate was compared with HRP by using the standard substrate of o-dianisidine. While the activity of HRP was determined as 117.876 U/mg, conjugate’s activity was found as 107.610 U/mg in which the relative activity of the conjugate was 91%. In the study of Lopes et al., the effect of microwave and conventional heating on the stability and structure of HRP was investigated and it was revealed that the activity of HRP reduced to 38% after 30 min of 60 W microwave irradiation at 45 °C [13]. Moreover, 30 min of microwave irradiation of HRP with 60 W of energy at 60 °C caused a decrease in the relative activity to 16.9%. It was concluded that microwave irradiation disrupts the tertiary structure of the enzyme, and it is a suitable method to inactivate the enzymes but, not an appropriate technique to be used in enzyme reactions. Sun et al. conjugated dextran with ovalbumin using single mode microwave heating [30]. The study reveals that microwave irradiation significantly increased the yield of the glycation reaction of ovalbumin with dextran to form conjugates. In addition, ovalbumin’s activity was increased after conjugation with dextran using microwave irradiation. It is concluded in the study that microwave irradiation is a safe method for bioconjugation. Our study follows the study of Sun et al. in which the relative activity of the HRP/D-CHO conjugate was almost the same as the free enzyme with decreasing only 9% in our study. The D-CHO conjugation of the enzyme prevented significant changes in the activity of HRP. This can be due to the multiple point conjugation of D-CHO on the surface of HRP and locking the 3D structure which may prevent changes in the secondary or tertiary structure of the enzyme. It is important to note that the microwave energy applied to the HRP/D-CHO conjugate in our study was much higher than studies of Lopes et al. The accelerating effects of microwave irradiation has recently been exhibited in the literature [13, 30]. In microwave heating, radiation is absorbed by the whole material, not only from the surface. Therefore, increasing the irradiation energy causes a higher energy transfer to the material and accelerates the reaction.

Since HRP is an industrially important enzyme that can be used in wastewater treatment, further experiments were focused on the decolorization of a toxic textile fiber dye of RB19. RB19 was chosen as a model synthetic textile dye because of its wide usage in the textile fiber industry. Decolorization of RB19 with HRP and HRP/D-CHO conjugate was carried out at different temperatures (25–50 °C).

**Figure 2:**
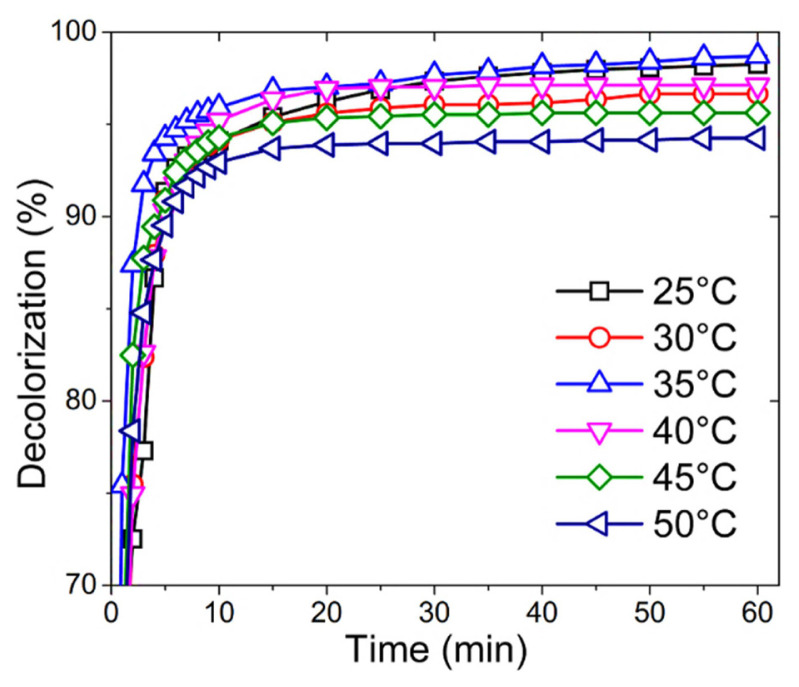
Decolorization of RB19 with HRP at different temperatures (25–50 °C) and pH 5.0.

Figure 2 shows the decolorization kinetics of RB19 with HRP as a function of temperature at pH 5.0. As seen, more than 90% of RB19 is decolorized in the first 5 min of the reaction at all temperatures. The decolorization of the dye reached to a maximum at 35 °C. Above 35 °C, decolorization of RB19 decreases in correlation with the temperature, in which the lowest value of decolorization was obtained at 50 °C. However, it is noteworthy that 94% of decolorization was reached even at 50 °C.

**Figure 3:**
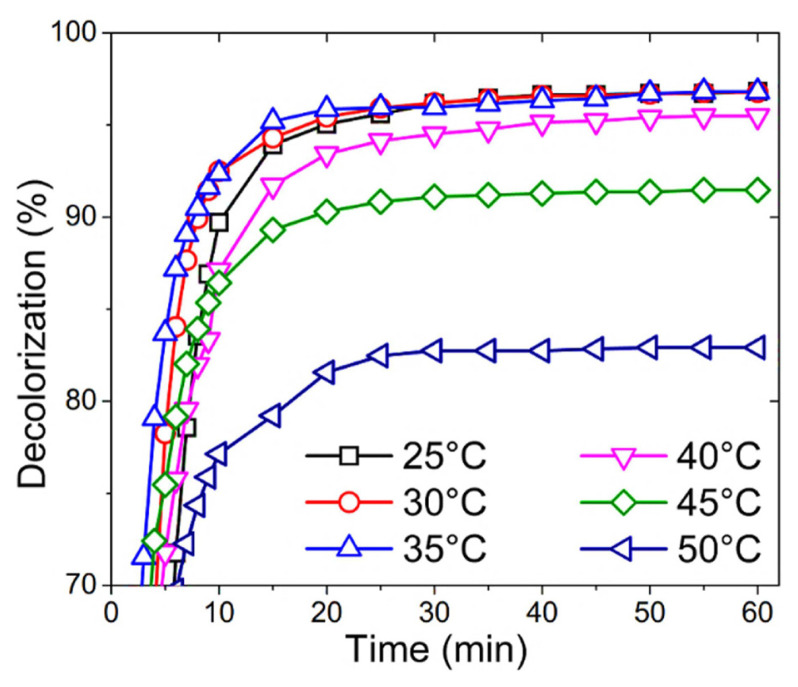
Decolorization of RB19 with HRP/D-CHO conjugate at different temperatures (25–50 °C) and pH 5.0.

Figure 3 belongs to the decolorization of RB19 with HRP/D-CHO conjugate. The maximum decolorization was obtained at 35 °C with the conjugate which is almost like the enzyme. However, there are significant differences between the enzyme and the conjugate’s activity in the decolorization of RB19. First, the decolorization reaction with the conjugate is slower than the enzyme, because in all temperatures maximum decolorization values were reached in longer times. Moreover, decolorization values were lower than the enzyme at 40, 45, and 50 °C, but decolorization did not decrease below 80% even at 50 °C.

In the literature, activities of purified HRP and its conjugate with D-CHO were investigated and conjugate’s activity was found to be lower than that of the pure enzyme [26]. On the other hand, the activity of HRP increased at a wider range of temperature and pH values after being immobilized in a hydrogel [22]. Therefore, it can be speculated that the change in the activity of HRP after modification may be related to modification type and modified group on the enzyme. In our study, it was observed that the activity of the enzyme-polymer conjugate is lower than the free enzyme but, it is important to indicate that the HRP-polymer conjugate still has a significant level of activity, especially at lower temperatures. This result reveals clearly that microwave irradiation can be used safely to produce enzyme-polymer conjugates efficiently in a much shorter time compared to the conventional heating process.

## 4. Discussion

We investigated the effect of microwave irradiation on the bioconjugation of HRP with D-CHO and the enzymatic activity of the conjugate. HRP and D-CHO were successfully conjugated with microwave heating in 5 min with a 75% yield. HRP exhibited its activity even after microwave-assisted conjugation with D-CHO. It is evident that D-CHO prevented the enzyme to lose its activity during the microwave irradiation and the produced conjugate still exposed at least 80% of decolorization activity compared to the free enzyme.

We consider that microwave irradiation can be used safely to obtain active enzyme-polymer conjugates for industrial applications. It is foreseen that the microwave-assisted synthesis described in the study may significantly improve the biopolymer-enzyme conjugation and immobilization processes.

## References

[1] Borah P Borah JM Chowdhury P Microwave (MW) irradiated Ugi four-component reaction (Ugi-4CR): Expedited synthesis of steroid-amino acid conjugates - A novel class of hybrid compounds Steroids 2015 98 49 57 10.1016/j.steroids.2015.02.012 25701096

[2] Chikhacheva IP Zubov VP Maslova EA Kurbakova IV Synthesis of polyacetals under microwave irradiation Russian Journal of Applied Chemistry 2008 81 1842 1845 10.1134/S107042720810025X

[3] Mamidala S Peddi SR Aravilli RK Jilloju PC Manga V Microwave irradiated one pot, three component synthesis of a new series of hybrid coumarin based thiazoles: Antibacterial evaluation and molecular docking studies Journal of Molecular Structure 2021 10.1016/j.molstruc.2020.129114

[4] Wang YM Zheng SX Chang HI Tsai HY Liang M Microwave-assisted synthesis of thermo- and pH-responsive antitumor drug carrier through reversible addition–fragmentation chain transfer polymerization Express Polymer Letters 2017 10.3144/expresspolymlett.2017.29

[5] Bhuiyan MMH Matin MM Bithi UH Alam MR Alam MA Solvent-free efficient microwave assisted synthesis of α, β-unsaturated compounds and their antimicrobial activity assessment Frontiers in Drug, Chemistry and Clinical Research 2019 2 1 6 10.15761/fdccr.1000131

[6] Güngör T Microwave assisted, sequential two-step, one-pot synthesis of novel imidazo [1,2-a] pyrimidine containing tri/tetrasubstituted imidazole derivatives Turkish Journal of Chemistry 2021 45 219 230 10.3906/KIM-2009-40 33679165 PMC7925308

[7] Wiesbrock F Hoogenboom R Leenen M VanNispen SFGM Van Der Loop M Microwave-Assisted Synthesis of a 4, 2 -Membered Library of Diblock Copoly (2-oxazoline) s and Chain-Extended Homo Poly (2-oxazoline) s and Their Thermal Characterization Macromolecules 2005 38 7957 7966 10.1021/ma050437x

[8] Amariucai-Mantu D Mangalagiu V Danac R Mangalagiu II Microwave assisted reactions of azaheterocycles formedicinal chemistry applications Molecules 2020 25 10.3390/molecules25030716 32046020 PMC7038048

[9] Banerjee J Hanson AJ Muhonen WW Shabb JB Mallik S Microwave-assisted synthesis of triple-helical, collagen-mimetic lipopeptides Nature Protocols 2010 5 39 50 10.1038/nprot.2009.195 20057380 PMC5826546

[10] Su Y Chen Z Li J Chang C Gu L Yang Y Characterization of salted egg yolk flavoring prepared by enzyme hydrolysis and microwave irradiation Food Chemistry 2021 10.1016/j.foodchem.2020.127913 33092000

[11] Roy I Gupta MN Applications of microwaves in biological sciences Current Science 2003 85 1685 1693

[12] Rejasse B Lamare S Legoy MD Besson T Influence of microwave irradiation on ezymatic properties: Applications in enzyme chemistry Journal of Enzyme Inhibition and Medicinal Chemistry 2007 22 519 527 10.1080/14756360701424959 18035819

[13] Lopes LC Barreto MTM Gonçalves KM Alvarez HM Heredia MF Stability and structural changes of horseradish peroxidase: Microwave versus conventional heating treatment Enzyme and Microbial Technology 2015 69 10 18 10.1016/j.enzmictec.2014.11.002 25640719

[14] Uchida T Sasaki M Tanaka Y Ishimori KA Dye-Decolorizing Peroxidase from Vibrio cholerae Biochemistry 2015 10.1021/acs.biochem.5b00952 26431465

[15] Šekuljica N Prlainović N Stefanović AB Žuža MG Čičkarić DZ Decolorization of anthraquinonic dyes from textile effluent using horseradish peroxidase: Optimization and kinetic study Scientific World Journal 2015 2015 10.1155/2015/371625 PMC4313523 25685837

[16] Altikatoglu M Arioz C Basaran Y Kuzu H Stabilization of horseradish peroxidase by covalent conjugation with dextran aldehyde against temperature and pH changes Open Chemistry 2009 7 423 428 10.2478/s11532-009-0041-z

[17] Melik-Nubarov NS Mozhaev VV Šikšnis S , Martinek S. Protein stabilization via hydrophilization: Stabilization of α-chymotrypsin by reductive alkylation with glyoxylic acid Biotechnology Letters 1987 9 725 730 10.1007/BF01024608

[18] Shmanai VV Litoshko AA Solid macrosupports for immunoassay, modified with polysaccharides Russian Journal of Applied Chemistry 2001 10.1023/A:1013770732638

[19] Bildirir H Effect of crosslinking patterns on the properties of conjugated microporouspolymers Turkish Journal of Chemistry 2019 43 730 739 10.3906/kim-1808-12

[20] Sentürk N Top A PEG-peptide conjugate containing cathepsin B degradation unit as a doxorubicin carrier system Turkish Journal of Chemistry 2018 42 385 400 10.3906/kim-1706-65

[21] Pasut G Polymers for protein conjugation Polymers 2014 6 160 178 10.3390/polym6010160

[22] Bilal M Rasheed T Zhao Y Iqbal HMN Agarose-chitosan hydrogel-immobilized horseradish peroxidase with sustainable bio-catalytic and dye degradation properties International Journal of Biological Macromolecules 2019 124 742 749 10.1016/j.ijbiomac.2018.11.220 30496859

[23] Siddique MH St Pierre CC Biswas N Bewtra JK Taylor KE Immobilized enzyme catalyzed removal of 4-chlorophenol from aqueous solution Water Research 1993 27 883 890 10.1016/0043-1354(93)90153-9

[24] Ihsanullah I Jamal A Ilyas M Zubair M Khan G Atieh MA Bioremediation of dyes: Current status and prospects Journal of Water Process Engineering 2020 38 101680 10.1016/j.jwpe.2020.101680

[25] Yuan H Chen L Cao Z Hong FF Enhanced decolourization efficiency of textile dye Reactive Blue 19 in a horizontal rotating reactor using strips of BNC-immobilized laccase: Optimization of conditions and comparison of decolourization efficiency Biochemical Engineering Journal 2020 156 107501 10.1016/j.bej.2020.107501

[26] Altikatoglu M Celebi M Enhanced stability and decolorization of Coomassie Brilliant Blue R-250 by dextran aldehyde-modified horseradish peroxidase Artificial Cells, Blood Substitutes, and Immobilization Biotechnology 2011 39 185 90 21117874 10.3109/10731199.2010.533124

[27] Altikatoglu M Arioz C Basaran Y Kuzu H Stabilization of horseradish peroxidase by covalent conjugation with dextran aldehyde against temperature and pH changes Open Chemistry 2009 7 423 428 10.2478/s11532-009-0041-z

[28] Cayir E Erdemir A Ozkan E Topuzogullari M Bolat ZB Cloning of intron-removed enolase gene and expression, purification, kinetic characterization of the enzyme from Theileria annulata Molecular Biotechnology 2014 56 689 96 10.1007/s12033-014-9747-z 24664479

[29] Celebi M Topuzogullari M Kuzu H Thermal Destabilization of Rhizomucor miehei Rennet with Aldehyde Dextran Sulfate: Purification, Bioconjugation and Milk-Clotting Activities Applied Biochemistry and Biotechnology 2016 180 261 273 10.1007/s12010-016-2097-5 27138725

[30] Sun J Mu Y Mohammed O Dong S Xu B Effects of single-mode microwave heating and dextran conjugation on the structure and functionality of ovalbumin–dextran conjugates Food Research International 2020 137 109468 10.1016/j.foodres.2020.109468 33233139

